# Preconditioning with Cathodal High-Definition Transcranial Direct Current Stimulation Sensitizes the Primary Motor Cortex to Subsequent Intermittent Theta Burst Stimulation

**DOI:** 10.1155/2021/8966584

**Published:** 2021-10-21

**Authors:** Wenjun Dai, Yao Geng, Hao Liu, Chuan Guo, Wenxiang Chen, Jinhui Ma, Jinjin Chen, Yanbing Jia, Ying Shen, Tong Wang

**Affiliations:** ^1^Rehabilitation Medicine Center, The First Affiliated Hospital of Nanjing Medical University, Nanjing, China; ^2^Neuro-Rehabilitation Center, JORU Rehabilitation Hospital, Yixing, China; ^3^Department of Rehabilitation, Children's Hospital of Nanjing Medical University, Nanjing, China; ^4^Department of Health Research Methods, Evidence, and Impact, McMaster University, Hamilton, ON, Canada

## Abstract

Noninvasive brain stimulation techniques such as transcranial magnetic stimulation (TMS) and transcranial direct current stimulation (tDCS) can induce long-term potentiation-like facilitation, but whether the combination of TMS and tDCS has additive effects is unclear. To address this issue, in this randomized crossover study, we investigated the effect of preconditioning with cathodal high-definition (HD) tDCS on intermittent theta burst stimulation- (iTBS-) induced plasticity in the left motor cortex. A total of 24 healthy volunteers received preconditioning with cathodal HD-tDCS or sham intervention prior to iTBS in a random order with a washout period of 1 week. The amplitude of motor evoked potentials (MEPs) was measured at baseline and at several time points (5, 10, 15, and 30 min) after iTBS to determine the effects of the intervention on cortical plasticity. Preconditioning with cathodal HD-tDCS followed by iTBS showed a greater increase in MEP amplitude than sham cathodal HD-tDCS preconditioning and iTBS at each time postintervention point, with longer-lasting after-effects on cortical excitability. These results demonstrate that preintervention with cathodal HD-tDCS primes the motor cortex for long-term potentiation induced by iTBS and is a potential strategy for improving the clinical outcome to guide therapeutic decisions.

## 1. Introduction

Noninvasive brain stimulation (NIBS) techniques such as transcranial magnetic stimulation (TMS) and transcranial direct current stimulation (tDCS) can induce a long-term potentiation- (LTP-) like facilitation or long-term depression- (LTD-) like suppression [1–3]. Depending on the stimulus frequency of repetitive (r)TMS or the polarity of electrodes placed on the scalp in tDCS, these 2 types of stimulation can bidirectionally regulate the excitability of neurons in neural networks [4–6]. Whether a greater effect can be achieved by combining TMS and tDCS is an open question.

Preconditioning with tDCS can alter the functional state of the motor cortex, resulting in TMS-induced changes in cortical plasticity [7–9]. When applied to the primary motor cortex (M1), inhibitory preconditioning with cathodal tDCS and subsequent 5-Hz rTMS significantly increased the excitability of the corticospinal tract, whereas facilitatory preconditioning with anodal tDCS and subsequent 5-Hz rTMS decreased excitability [7, 9]. The preconditioning effects of tDCS may be related to synaptic homeostatic plasticity which is thought to maintain neural activity within a certain physiological range and ensure the stability of neural network activity [10]. As described in the Bienenstock–Cooper–Munro (BCM) theory of bidirectional synaptic plasticity [11], it states that stable neuronal activity is achieved through dynamic regulation of the threshold of synaptic modification. The threshold is decreased by a reduction in postsynaptic activity, which can induce LTP, whereas increased postsynaptic activity raises the threshold and induces LTD. Thus, the state of synaptic activation before stimulation influences the after-effects of NIBS.

Intermittent theta burst stimulation (iTBS) activates N-methyl-d-aspartate (NMDA) receptor [12], shortens the time for therapy [13], and induces LTP-like when assessed in humans with brain stimulation [14]. Conventional tDCS modulates the excitability in the brain with constant, low-intensity direct current (1–2 mA) [15]. Anodal tDCS was shown to depolarize the membrane potential by activating Na^+^ and Ca^2+^ voltage-gated channels and increasing neuronal excitability and cortical activity, while cathodal tDCS caused the opposite effects by inducing membrane hyperpolarization [15, 16]. In clinical applications, conventional tDCS electrodes have the disadvantage of insufficient spatial resolution because of their large size (16–35 cm^2^). High-definition (HD) tDCS has improved resolution because it uses electrodes in a 4 × 1 ring configuration that is based on a high-resolution magnetic resonance imaging-based finite element model [17]. Given its unique advantages in regulating cortical excitability, the combined application of HD-tDCS and iTBS deserves further investigation.

Here, we hypothesized that preconditioning with cathodal HD-tDCS would sensitize the primary motor cortex to subsequent iTBS, and the after-effect would last longer compared to iTBS without the preconditioning in healthy young adults.

## 2. Materials and Methods

### 2.1. Participants

A total of 24 healthy volunteers (4 males, 20 females; mean age 21.29 ± 0.73 years) participated in the experiments (Table 1). Inclusion criteria were as follows: (1) no neurologic or psychiatric disorders or serious illnesses and (2) right-handed, as verified using the Edinburgh Handedness Inventory. Exclusion criteria were as follows: (1) a history of epilepsy, idiopathic epilepsy in a first-degree relative, and use of epileptogenic drugs and (2) implants including an artificial metal heart valve, insulin pump, drug treatment pump, or aneurysm clip (nonparamagnetic, except titanium alloy). All subjects participated in the study voluntarily and signed the written informed consent form. The study was approved by the Ethics Committee of JORU Rehabilitation Hospital (approval no. 20201130A02) and was registered with the China Clinical Trial Registration Center (http://www.chictr.org.cn; no. ChiCTR2000041144). All experimental procedures were carried out according to the principles outlined in the Declaration of Helsinki.

### 2.2. Experimental Design

**Figure 1:**
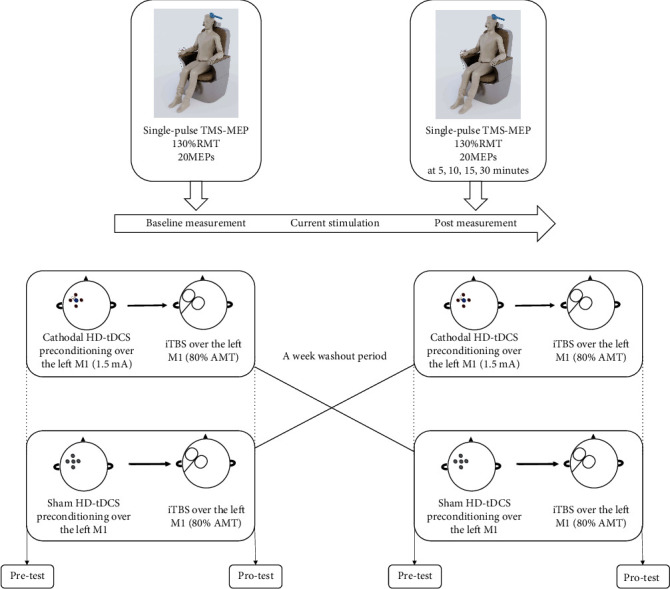
Experimental design. Single-pulse TMS was delivered to the left M1. The coil was first placed on the motor hotspot that produced an optimum response defined by electromyography recordings of abductor pollicis brevis (APB) muscle. Twenty MEPs induced at a TMS intensity of 130% resting motor threshold (RMT) were averaged as the baseline cortical excitability (a). MEPs were recorded in the pretest block. Subjects received twenty consecutive single pulses over the target site of the left M1 with an interval of 5 s. Preconditioning with cathodal HD-tDCS or sham prior to iTBS was applied. One week later, the interventions were switched (b). The after-effects were measured as the amplitude of MEPs at 5, 10, 15, and 30 min after the intervention. AMT: active motor threshold.

This study adopted a randomized crossover design (Figure 1). All subjects were randomized to receive preconditioning with cathodal HD-tDCS or sham cathodal HD-tDCS prior to iTBS with a washout period of 1 week. We measured the amplitude of MEPs at baseline and at different time points (5, 10, 15, and 30 min) after the intervention.

### 2.3. Assessment

#### 2.3.1. Determination of Motor Hotspots

The experiment was conducted in a quiet isolated room. During the experiment, subjects were prohibited from talking, sleeping, and using their mobile phones. The subjects were comfortably seated on a reclining chair with neck, arms, and legs supported in a relaxed position and were asked to wear a positioning scalp cap to reduce sliding between their hair and the stimulus coil. The center of the coil was placed tangentially on the scalp over the motor hotspot for the right abductor pollicis brevis (AFB) that was marked beforehand, with the handle posterior to the midline at an angle of 45°. The surface electromyography (sEMG) recording electrode was placed on the APB of the right hand and MEPs were recorded using the CCY-I TMS system (Yiruide, Wuhan, China) and were analyzed with the accompanying software. The optimal scalp location was the one that produced the maximum response in EMG recordings of the APB. The motor hotspot was marked on the scalp for reference and was continually monitored throughout the experiment.

#### 2.3.2. Measurement of Cortical Plasticity

Cortical plasticity was assessed by investigating changes in the amplitude of MEPs in the left M1 recorded during complete relaxation of the APB of the right hand. Twenty consecutive MEPs (5 s interval) were evoked by single-pulse TMS with an intensity of 130% resting motor threshold (RMT). RMT was defined as the lowest stimulation intensity to evoke an MEP > 50 *μ*V in the relaxed APB for at least 5 of 10 consecutive TMS pulses [18, 19]. MEP amplitudes were extracted and calculated as peak-to-peak amplitudes of trials, without elimination of the maximum and minimum MEP amplitudes. The peak-to-peak value was averaged as the baseline. Two baseline measurements were obtained (separated by 5 min) before any intervention in order to assure the intraindividual reliability of cortical excitability [20], and the plasticity protocol was applied if there was no more than a 10% difference between the 2 baseline values.

### 2.4. Interventions

#### 2.4.1. Cathodal HD-tDCS

The device used a 4 × 1 HD-tDCS adaptor (Soterix Medical, New York, NY, USA). Five small circular electrodes (l cm^2^) replaced the traditional large sponge electrode. The central cathodal electrode was placed on the scalp above the left M1 where the motor hotspot was previously marked, and the 4 return electrodes (separated from the central electrode by a distance of 3.5 cm) were placed so as to form a circular current loop after applying conductive paste. The tDCS was delivered at a current intensity of 1.5 mA for 20 min after checking the connection quality. For the sham protocol, the current was increased slowly to 1.5 mA in the first 15 s and then gradually decreased to 0 in 15 s; the other settings were the same as for the real stimulation.

#### 2.4.2. iTBS

iTBS was performed with a CCY-I TMS stimulator using a figure 8-shaped coil for accurately targeted stimulation. The magnetic stimulus had a biphasic waveform. It can produce a maximum stimulator output (MSO) of 3.0 Tesla [21]. The iTBS pattern, which has been described in a previous study [14], consisted of bursts of 3 pulses at 50 Hz repeated at 5 Hz; a 2 s train of TBS was repeated every 10 s for a total of 192 s (600 pulses). The stimulation intensity of the experiment was 80% of the active motor threshold. iTBS was delivered over the left motor hotspot to modulate cortical plasticity.

### 2.5. Data Analysis

A total of 24 subjects were required to detect a standardized effect size of 0.8 (cathodal HD-tDCS+iTBS vs. sham cathodal HD-tDCS+iTBS) based on data from our pilot study with a statistical power of 80% at the significance level of 0.01 (2-sided test).

Both raw MEP amplitude and normalized MEP were analyzed. Normalized MEPs were calculated as raw MEP amplitude divided by the mean of MEP at baseline in the same intervention group and presented in percentage. MEP data at each time point were summarized as mean and standard deviation (SD). The MEP data were analyzed using two-way repeated-measures analysis of variance (ANOVA). A *p* value < 0.05 was considered statistically significant. Since the interaction between time and intervention group was statistically significant, we further estimated the within- and between-group difference and the corresponding 95% confidence interval (CI).

## 3. Results

All 24 subjects participated in 2 intervention sessions.  Table 1 shows the demographic characteristics of the subjects. Twelve subjects (including 3 males) received cathodal HD-tDCS+iTBS in the first session, and the others (including 1 male) were assigned to the sham cathodal HD-tDCS+iTBS group. The experimental procedure was well tolerated and none of the subjects experienced any adverse effects (e.g., headache, giddiness, and fidgeting) during or after the intervention.

**Figure 2:**
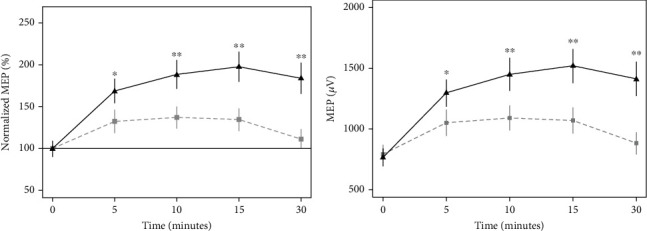
Time course of corticospinal excitability. The grey square represents mean MEP amplitude for subjects receiving sham cathodal HD-tDCS and iTBS. The black triangle represents mean MEP amplitude for subjects receiving cathodal HD-tDCS and iTBS. Vertical lines indicate standard error of the mean of MEP amplitudes. (a) Shows the normalized MEP (%) over time. (b) Shows MEP (*μ*V) over time. ∗ indicates that the between-group difference is significant with 0.001 < *p* < 0.05. ∗∗ indicates the between-group difference is significant with *p* < 0.001.

The MEP measures at baseline before and after the crossover were compared, and there were no significant differences (Table 2). In addition, the MEP measures at different time points are presented in Table 3. Preconditioning with cathodal HD-tDCS and iTBS had a marked effect on cortical excitability. Subjects showed an increase in MEP amplitude at 5 min postintervention compared to the respective baseline values in both intervention groups (increase = 258 *μ*V, 95%CI = (100, 417), *p* < 0.001 for the sham group; increase = 529 *μ*V, 95%CI = (371, 687), *p* < 0.001 for the cathodal HD-tDCS prior to iTBS group). The MEP amplitude in subjects receiving cathodal HD-tDCS prior to iTBS increased continuously up to 15 min and then decreased slightly thereafter; by 30 min, MEP amplitude was still greater than at baseline (increase = 645 *μ*V, 95%CI = (487, 803); *p* < 0.001). In contrast, in subjects receiving the sham intervention, MEP amplitude dropped slightly between 5 and 15 min and decreased substantially by 30 minutes. The results from the repeated-measures ANOVA on raw MEP showed that cathodal HD-tDCS and iTBS increased MEP amplitude to a greater extent than the sham intervention overall (*F*(1, 207) = 75.3, *p* < 0.001 for intervention; *F* (4, 207) = 26.53, *p* < 0.001 for time; and *F*(4, 207) = 7.23, *p* < 0.001 for intervention and time interaction). Results from the same analysis on normalized MEP were similar to those on raw MEP. These results indicate that cathodal HD-tDCS enhances the effects of iTBS as evidenced by increased MEP amplitude compared to iTBS without the preconditioning and that the effect is long-lasting. Detailed results from two-way repeated-measures ANOVA are presented in Table 4 and Figure 2.

## 4. Discussion

The combination of tDCS and TMS is increasingly being applied in clinical and research settings to induce LTP-like and LTD-like plasticity [7, 8, 10]. There have been few studies to date investigating whether preconditioning with HD-tDCS can enhance the effect of iTBS in healthy subjects and patients.

In this study, we found that the cortical excitability of M1 was increased with iTBS after inhibitory preconditioning by cathodal HD-tDCS compared to sham HD-tDCS; MEP amplitudes at nearly all postintervention time points showed obvious changes, with the effects lasting for 5–30 min. This is in agreement with previous studies demonstrating that a preconditioning protocol with cathodal tDCS potentiated cortical plasticity induced by 5 Hz rTMS [7] and altered the baseline state of motor cortical excitability, thereby reversing or enhancing the after-effects of rTMS [8].

In contrast to previous studies, we used HD-tDCS for preconditioning. The central electrode was placed at the stimulation target surrounded by 4 return electrodes; the 4 × 1 ring configuration of the electrodes was effective in inducing plasticity in M1 [22, 23]. Conventional tDCS delivers diffuse current to the cerebral cortex, making it difficult to establish a causal relationship between cortical stimulation and behavioral changes [24]. In contrast, the electrode placement for HD-tDCS creates a peak induction field under the active electrode, and the distance between the active and return electrodes limits the spatial distribution of the electric field and current delivered to the brain, thereby improving current stimulation focality [17, 25]. Additionally, HD-tDCS achieves a longer-lasting after-effect than conventional tDCS [23]. We used iTBS for the subsequent test NIBS. Compared with rTMS, iTBS imitates endogenous theta rhythms, which results in better induction of synaptic LTP [26]. iTBS intervention, as an extremely efficient and useful protocol for basic and clinical applications, enables the delivered time shorter than standard rTMS protocols [13]. Also, the most distinguished originality in our research is that the application of iTBS follows with HD-tDCS immediately. Lang et al. found that the combination of cathodal tDCS and 5 Hz rTMS with an interval of 10 minutes increased MEP amplitude [7], while the combination of anodal tDCS and 1 Hz rTMS with an interval of 10 minutes decreased MEP amplitude [8].

All in all, compared with previous studies about combined intervention of rTMS and tDCS, iTBS shortened the duration of intervention. In addition, preconditioning with cathodal HD-tDCS followed by iTBS without time intervals could also induce LTP-like plasticity.

The effect of preconditioning can be explained by the mechanism of synaptic homeostatic plasticity [7, 8]. The induction and direction of synaptic plasticity depend on the excitability of the postsynaptic neuron at the time of stimulation [27], which is significant for the stability stable neuronal networks [28, 29]. During the learning and development processes of the brain, synaptic excitability is modulated by homeostasis [30], which depends on intracellular Ca^2+^ level; the firing rate remains relatively constant, and neural network activity is stable [29]. With changes in neural activity and intracellular Ca^2+^ level, the firing rate of neurons is stabilized through an increase or decrease in synaptic strength, thus ensuring normal information processing and storage [29, 31].

Studies have indicated that the combined application of various NIBS procedures could induce synaptic homeostatic plasticity [10]. A preconditioning NIBS procedure could enhance the effect of a subsequent NIBS procedure with an opposing effect. In contrast, if the influence of the two procedures was the same, preconditioning with a NIBS procedure could weaken the response to a subsequent NIBS procedure [7–9]. According to the BCM theory of bidirectional synaptic plasticity, a high level of postsynaptic activity favors LTD whereas a low level of that induces LTP [11, 32]. Moreover, the theory proposes that the sliding threshold (i.e., the critical point at which LTD-like plasticity becomes LTP-like) is not fixed but is dependent on postsynaptic activity [10]. Application of LTD-like preconditioning lowers the sliding threshold, contributing to a decrease in postsynaptic activity and thereby facilitating LTP induction. Thus, homeostatic mechanisms compensate for decreased firing owing to a reduction in or loss of activity, leading to compensatory changes in excitatory synapses and improved synaptic efficiency.

Homeostatic plasticity of synapses has also been observed in animal experiments [33, 34]. By monitoring the response of extracellular excitatory postsynaptic potentials in the hippocampal CA1 stratum radiatum to Schaffer collateral stimulation, it was found that preconditioning applied to a specific synaptic pathway altered the effect of a subsequent stimulation protocol; for example, preconditioning with high-frequency stimulation increased neuronal excitability but the excitatory effect was reversed by stimulation at 10 Hz, resulting in LTD induction [34].

Some researches also illustrated that the interval between the two NIBS protocols could influence the direction of cortical excitability affected by the subsequent NIBS intervention [35, 36]. It is important for deciding the direction of subsequent plasticity; however, the direction of response can be variable. Tse et al. found that a shorter interval of 5 minutes could invert cortical excitability, while a longer interval of 15 minutes could enhance the after effects induced by preconditioning, which led to a potentiation compared with a single stimulation [36]. According to the existing researches, the results indicated that there was LTD-like effect in the brain cortex after preconditioning with cathodal HD-tDCS [15, 16]. Based on synaptic homeostatic plasticity, cathodal HD-tDCS reduced the average postsynaptic activity in M1, decreasing the threshold for LTP induction. Subsequent iTBS, which had the opposite effect to HD-tDCS, resulted in prolonged excitability of the corticospinal tract.

Although we demonstrated the effectiveness of the combined NIBS procedure, the safety and tolerability of TMS and tDCS are important considerations. The symptoms of headache, tingling, and itching associated with TMS or tDCS are usually mild and short-lived [37–40], but few studies have systematically investigated the tolerability of these procedures, which has prevented the widespread application of NIBS to the intervention of neurologic or psychiatric disorders. No serious adverse events occurred during the course of the present study, and there was no increase in the risk of intervention-related adverse events following the intervention. In the future, we will review the safety of the protocol in a larger sample.

Preconditioning with cathodal HD-tDCS enhanced iTBS-induced cortical plasticity in the M1 area of the brain, and its duration of after-effect was prolonged. We aimed to search for a novel pattern by which the excitability of cerebral cortex could be better and steadily regulated. This preliminary study provides a basis for investigating the best stimulation parameters of this combined intervention in the future. Additionally, these unclear factors need to be verified in further relevant RCTs, which may be the existence or nonexistence of the synaptic homeostatic plasticity mechanism in people of different ages and diseases, the safety of the combination of tDCS and TMS, and the effect of different intervals between the two NIBS interventions.

## 5. Conclusions

In conclusion, cathodal HD-tDCS followed by iTBS in M1 increased MEP amplitude and enhanced cortical plasticity with long-lasting effects compared to iTBS without HD-tDCS preconditioning in healthy young adults. These results suggest that preconditioning with cathodal HD-tDCS can be used to improve the therapeutic efficacy of iTBS and have prolonged after-effects, which has a high potential for clinical application and deserves to be explored in further studies.

## Figures and Tables

**Table 1:** Characteristics of the study subjects.

Characteristics	Results (*n* = 24)
Age (years), mean (SD)	21.3 (0.7)
Female, *n* (%)	20 (83.3%)
Hand of preference (right hand), *n* (%)	24 (100%)
RMT (%MSO), mean (SD)	46.3 (8.8)

SD: standard deviation; RMT: resting motor threshold; MSO: maximal stimulator output.

**Table 2:** Summary of MEP amplitudes at baseline.

Subjects	Before crossover mean (SD)	After crossover mean (SD)	*p* value
Sham➔cathodal HD-tDCS+iTBS (*n* = 12)	721 (294)	718 (300)	0.83
Cathodal➔sham HD-tDCS+iTBS (*n* = 12)	817 (408)	862 (437)	0.14
All (*n* = 24)	769 (351)	790 (373)	0.24

MEP: motor evoked potential; SD: standard deviation, ➔: crossover from … to ….

**Table 3:** Summary of MEP amplitudes at different time points.

Time	Sham cathodal HD-tDCS+iTBS mean (SD)	Cathodal HD-tDCS+iTBS mean (SD)
Raw MEP (*μ*V)		
Baseline	792 (372)	767 (354)
5 minutes	1051 (528)	1296 (567)
10 minutes	1090 (501)	1448 (654)
15 minutes	1068 (522)	1518 (681)
30 minutes	883 (446)	1412 (692)
Normalized MEP (%)		
Baseline	100 (47)	100 (46)
5 minutes	133 (67)	169 (70)
10 minutes	138 (63)	189 (85)
15 minutes	135 (66)	198 (89)
30 minutes	112 (56)	184 (90)

MEP: motor evoked potential; SD: standard deviation.

**Table 4:** Comparison of MEP at different intervention groups and at different time points.

Time	Within-group change (95% CI)		Between-group difference in change (95% CI)
	Sham cathodal HD-tDCS+iTBS	Cathodal HD-tDCS+iTBS	
Raw MEP (*μ*V)			
From 5 min to baseline	258 (100, 417)^∗∗^	529 (371, 687)^∗∗^	270 (47, 494)^∗^
From 10 min to baseline	297 (140, 456)^∗∗^	680 (522, 839)^∗∗^	383 (159, 606)^∗∗^
From 15 min to baseline	276 (118, 434)^∗∗^	750 (593, 909)^∗∗^	475 (251, 698)^∗∗^
From 30 min to baseline	91 (-67, 249)	645 (487, 803)^∗∗^	554 (330, 777)^∗∗^
Normalized MEP (%)			
From 5 min to baseline	33 (12, 53)^∗∗^	69 (48, 89)^∗∗^	36 (7, 65)^∗^
From 10 min to baseline	38 (17, 58)^∗∗^	89 (68, 109)^∗∗^	51 (22, 80)^∗∗^
From 15 min to baseline	35 (14, 55)^∗∗^	98 (77, 118)^∗∗^	63 (34, 92)^∗∗^
From 30 min to baseline	12 (-9, 32)	84 (63, 105)^∗∗^	73 (44, 102)^∗∗^

MEP: motor evoked potential; CI: confidence interval; ^∗^0.01 < p < 0.05; ^∗∗^*p* < 0.001.

## Data Availability

The data presented in this article can be obtained from the corresponding author on reasonable request.

## Supplementary Materials

Supplementary Materials We describe raw MEP amplitudes during the pilot study in Supplementary Table 1.
