# Supplementary material for: Preconditioning with Cathodal High-Definition Transcranial Direct Current Stimulation Sensitizes the Primary Motor Cortex to Subsequent Intermittent Theta Burst Stimulation
Source: Neural Plast. 2021 Oct 21;2021:8966584. doi: 10.1155/2021/8966584 (PMC8553444; doi:10.1155/2021/8966584)
Supplement: Supplementary Materials — We describe raw MEP amplitudes during the pilot study in Supplementary Table 1. [file 8966584.f1.pdf]

Supplementary Table 1: Raw MEP amplitudes during the pilot study

			Cathodal HD-tDCS+iTBS MEP amplitude （μV）						Washout period（1 week）	Sham HD-tDCS+iTBS MEP amplitude （μV）					
Subjects	Age(years)	Gender	Baseline1	Baseline2	5min	10min	15min	30min		Baseline1	Baseline2	5min	10min	15min	30min
1	23	F	527	493	684	925	1242	936		592	540	556	922	847	856
2	21	F	689	652	1089	1059	1076	1427		714	676	846	1073	987	1025
3	21	F	785	733	1288	1197	1457	1264		796	740	1069	1275	1098	883
4	20	F	762	710	1497	1359	1532	876		737	681	1078	802	1224	966
5	21	M	742	723	1071	756	1673	1223		726	696	798	831	822	948
			Sham HD-tDCS+iTBS MEP amplitude （μV）							Cathodal HD-tDCS+iTBS MEP amplitude （μV）					
			Baseline1	Baseline2	5min	10min	15min	30min		Baseline1	Baseline2	5min	10min	15min	30min
6	22	F	812	885	1299	1449	1545	819		755	810	1497	1359	1665	683
7	21	F	626	585	681	994	1242	828		687	633	1464	1284	1287	1069
8	22	F	512	480	544	548	606	500		496	468	966	1020	942	1038
9	20	F	728	783	993	1074	639	1053		713	768	1782	1296	1437	1332
10	21	M	619	584	734	639	620	446		563	534	657	611	704	499

MEP = motor evoked potential
